# Comparable Immune Alterations and Inflammatory Signatures in ME/CFS and Long COVID

**DOI:** 10.3390/biomedicines13123001

**Published:** 2025-12-08

**Authors:** Steliyan Petrov, Martina Bozhkova, Mariya Ivanovska, Teodora Kalfova, Dobrina Dudova, Ralitsa Nikolova, Katya Vaseva, Yana Todorova, Milena Aleksova, Maria Nikolova, Hristo Taskov, Marianna Murdjeva, Michael Maes

**Affiliations:** 1 Department of Medical Microbiology and Immunology “Prof. Dr. Elissay Yanev”, Medical University of Plovdiv, 4002 Plovdiv, Bulgaria; martina.bozhkova@mu-plovdiv.bg (M.B.); mariya.ivanovska@mu-plovdiv.bg (M.I.); teodora.kalfova@mu-plovdiv.bg (T.K.); dobrina.dudova@mu-plovdiv.bg (D.D.); ralitsa.nikolova@mu-plovdiv.bg (R.N.); katya.vaseva@mu-plovdiv.bg (K.V.); hristo.taskov@mu-plovdiv.bg (H.T.); 2 National Reference Laboratory of Immunology, National Center of Infectious and Parasitic Diseases (NCIPD), 1504 Sofia, Bulgaria; y_todorova@abv.bg (Y.T.); milena.aleksova@hotmail.com (M.A.); mr_nklv@yahoo.com (M.N.); 3 Department of Microbiology and Virology, Medical University of Pleven, 5800 Pleven, Bulgaria; mmurdjeva@yahoo.com; 4 Institute of Innovation and Smart Technology, University of Telecommunication and Posts, 1700 Sofia, Bulgaria; 5 Sichuan Provincial Center for Mental Health, Sichuan Provincial People’s Hospital, School of Medicine, University of Electronic Science and Technology of China, Chengdu 610072, China; dr.michaelmaes@hotmail.com; 6 Key Laboratory of Psychosomatic Medicine, Chinese Academy of Medical Sciences, Chengdu 610072, China; 7 Department of Psychiatry, Medical University of Plovdiv, 4002 Plovdiv, Bulgaria; 8 Research Institute, Medical University of Plovdiv, 4002 Plovdiv, Bulgaria; 9 Research and Innovation Program for the Development of MU-PLOVDIV (SRIPD-MUP), Creation of a Network of Research Higher Schools, National Plan for Recovery and Sustainability, European Union–NextGenerationEU, Medical University of Plovdiv, 4002 Plovdiv, Bulgaria

**Keywords:** COVID-19, long COVID, chronic fatigue syndrome, chronic fatigue

## Abstract

**Background:** Chronic Fatigue Syndrome (CFS), also known as Myalgic Encephalomyelitis (ME), is a debilitating condition characterized by persistent fatigue and multisystemic symptoms, such as cognitive impairment, musculoskeletal pain, and post-exertional malaise. Recently, parallels have been drawn between ME/CFS and Long COVID, a post-viral syndrome following infection with SARS-CoV-2, which shares many clinical features with CFS. Both conditions involve chronic immune activation, raising questions about their immunopathological overlap. **Objectives:** This study aimed to compare immune biomarkers between patients with ME/CFS or Long COVID and healthy controls to explore shared immune dysfunction. **Methods:** We analyzed lymphocyte subsets, cytokine profiles, psychological status and their correlations in 190 participants, 65 with CFS, 54 with Long COVID, and 70 healthy controls. **Results:** When compared to healthy subjects, results in both conditions were marked by lower levels of lymphocytes (CFS—2.472 × 10^9^/L, *p* = 0.006, LC—2.051 × 10^9^/L, *p* = 0.009), CD8^+^ T cells (CFS—0.394 × 10^9^/L, *p* = 0.001, LC—0.404 × 10^9^/L, *p* = 0.001), and NK cells (CFS—0.205 × 10^9^/L, *p* = 0.001, LC—0.180 × 10^9^/L, *p* = 0.001), and higher levels of proinflammatory cytokines such as IL-6 (CFS—3.35 pg/mL, *p* = 0.050 LC—4.04 pg/mL, *p* = 0.001), TNF (CFS—2.64 pg/mL, *p* = 0.023, LC—2.50 pg/mL, *p* = 0.025), IL-4 (CFS—3.72 pg/mL, *p* = 0.041, LC—3.45 pg/mL, *p* = 0.048), and IL-10 (CFS—2.29 pg/mL, *p* = 0.039, LC—2.25 pg/mL, *p* = 0.018). **Conclusions:** Notably, there were no significant differences between CFS and Long COVID patients in the tested biomarkers. These results demonstrate that ME/CFS and Long COVID display comparable immune and inflammatory profiles, with no significant biomarker differences observed between the two groups.

## 1. Introduction

COVID-19, caused by the SARS-CoV-2 virus, was declared a pandemic by the WHO in March 2020 [1] and resulted in millions of deaths. Symptoms vary widely, from asymptomatic cases to respiratory signs like fever, cough, and shortness of breath, alongside systemic effects such as gastrointestinal and neurological complications [2,3,4,5]. While much focus has been on acute symptoms, interest in long-term effects, including “long COVID,” is growing. Long COVID has been linked to myalgic encephalomyelitis/chronic fatigue syndrome (ME/CFS), a chronic illness marked by at least six months of fatigue [6]. Controversy surrounds ME/CFS due to the absence of clear diagnostic tests and its reliance on subjective reports, leading to stigma. ME/CFS affects more women than men and was previously dismissed as “Yuppie Flu” [7]. Research shows that ME/CFS symptoms like fatigue, pain, and cognitive issues worsen over time, with few patients achieving remission. The most common hypothetical trigger for the illness is an infectious event, leading to a gradual worsening of symptoms [8]. In the course of time, several hypotheses have been proposed to explain the pathophysiology of ME/CFS, with one of the most prominent theories involving infection with the Epstein–Barr virus (EBV), leading to chronic inflammatory state [9]. Not only can persistent infections create a chronic inflammatory state, but acute microbial infections can also lead to chronic systemic inflammation, including conditions such as enteroviruses, cytomegalovirus, human herpesvirus-6, human parvovirus B19, hepatitis C, Chlamydophila pneumoniae, and Coxiella burnetii [10].

A critical aspect of the immune dysfunction observed in both ME/CFS and Long COVID is the abnormal function and regulation of natural killer cells. NK cells play an irreplaceable role in the immune system and a decline in their function has been reported in the context of ME/CFS [11,12,13,14]. NK cells play a vital role in the immune system by identifying and destroying virally infected cells and tumor cells. Multiple studies have consistently reported decreased NK cell function in ME/CFS patients. Positive findings in this area have been documented in some studies [14,15,16,17]. However, a few studies did not observe this dysfunction, highlighting the complexity and potential heterogeneity of ME/CFS [18,19]. In contrast, research into Long COVID has shown mixed results. While some studies have reported NK cell abnormalities, others have not, indicating that NK cell dysfunction might not be as universally characteristic of Long COVID as it is of ME/CFS [20].

Cytokine imbalance may play a key role in the causes of ME/CFS [21]. Previous investigations have found increased blood levels of TGF-β1 in ME/CFS patients compared to controls [11,22], while other researchers report the opposite results [23]. ME/CFS patients show a Th2 profile of CD4-helper T-lymphocyte responsiveness, with lower IFN-γ (inhibitory pathway) production by CD4^+^ cells [12,21,24,25,26]. Skowera et al. [26] discovered a considerable increase in the number of CD4^+^ and CD8^+^ T cells secreting IL-4, both after polyclonal stimulation and in resting cell populations from ME/CFS patients.

Soon after the COVID-19 pandemic began, it became evident that some patients continued to experience health issues for months or even years after infection with SARS-CoV-2. This novel syndrome has been given many names, such as Post-Acute COVID Syndrome (PACS), Post-Acute Sequelae of COVID-19 (PASC), and long-haul COVID, but it is most widely recognized in the literature as Long COVID [27]. This condition is marked by the following most frequent symptoms: fatigue, dyspnoea, cognitive and mental impairment, headache, myalgia, chest and joint pains [28]. There are many hypotheses about the pathophysiology of this illness, including long lasting inflammation due to the dysregulation of the immune system, persisting reservoirs of SARS-CoV-2 in tissues, autoimmunity, endothelial dysfunction and disrupted signaling in the brainstem and/or vagus nerve [29,30].

There is now evidence that mood disorders including depression and anxiety have an organic substrate and are characterized by activated immune-inflammatory pathways [31], including increased levels of proinflammatory and anti-inflammatory cytokines. Therefore, it is relevant to propose that mood symptoms due to COVID-19 are mediated at least in part by neuro-immune pathways. People with COVID-19 also frequently suffer from mental fatigue, physical fatigue, mild loss of concentration, neurocognitive deficits, headache and myalgia [32], symptoms which are reminiscent of ME/CFS [33].

Although Long COVID and ME/CFS exhibit overlapping symptoms, the extent to which they share underlying immunological or psychological mechanisms remains unclear [34,35,36,37]. Existing studies typically focus on one condition in isolation, and direct comparative analyses are not very common. This gap hampers efforts to refine diagnostic criteria, understand disease trajectories, and identify shared paths. Therefore, we aimed to investigate and compare key immunological parameters and psychological status in individuals with ME/CFS and Long COVID.

## 2. Materials and Methods

### 2.1. Patient Characteristics

This study was conducted and reported following STROBE guidelines for observational research. The study included a total of 190 participants, divided into 3 subgroups: (i) CFS group (*n* = 65), consisting of 20 males (39.94 ± 2.80 y.o.) and 45 females (42.33 y.o. ± 1.79); (ii) Long COVID (LC) group (*n* = 54), consistng of 11 males (41.94 ± 2.39 y.o.) and 43 females (48.61 ± 1.84 y.o.); and (iii) healthy control (HC) group (*n* = 70), consisting of 27 males (25.41 ± 1.68 y.o.) and 43 females (30.69 ± 2.12 y.o.).

### 2.2. Inclusion Criteria

CFS patients were included in the study according to the diagnostic criteria proposed by the Institute of Medicine (IOM) in 2015, based on the following three symptoms: 1. Significant reduction or limitation in the ability to perform pre-illness levels of daily activities that persists for more than 6 months, accompanied by fatigue that is often profound, of recent or definite (not lifelong) onset, and is not substantially relieved by rest. 2. Post-exertional malaise. 3. Unrefreshing sleep. At least one of the following is also required—cognitive impairment or orthostatic intolerance [38]. Participants in the LC group were categorized as such, based on a positive SARS-CoV-2 PCR test and persistence of symptoms (fatigue/post-exertional malaise, dyspnoea, joint pain, cognitive impairment, sleep disturbances, cardiovascular and gastrointestinal symptoms) more than 4 months after recovery that cannot be explained by other conditions [28]. Healthy subjects were enrolled based on the absence of an active SARS-CoV-2 infection, confirmed by positive PCR/anti-SARS-CoV-2 IgG, and an absence of Long COVID-19/CFS symptoms.

### 2.3. Exclusion Criteria

We excluded pregnant and breastfeeding women. Subjects who suffered from psychiatric disorders such as major depressive disorder, generalized anxiety disorder, bipolar disorder, panic disorder, schizo-affective disorder, schizophrenia, psycho-organic syndrome, and substance use disorders (except tobacco use disorder (TUD)) were not included in the study. We also excluded patients with cancer, systemic autoimmune diseases such as rheumatoid arthritis, diabetes mellitus, psoriasis, inflammatory bowel disease, neurodegenerative diseases (e.g., multiple sclerosis, Parkinson’s or Alzheimer’s disease) and stroke, as well as those with renal and liver disease. All of the participants signed their written consent. The study was conducted in accordance with the Declaration of Helsinki, and approved by the Scientific Ethics Committee at the Medical University of Plovdiv, Protocol No. 3/30 May 2023, for studies involving humans.

### 2.4. Clinical Examination

The participants’ health status, including their physical, mental, and behavioral well-being, were assessed by an experienced team of immunology specialists using a methodical interview. Sociodemographic, clinical, and psychological details were gathered through semi-structured interviews. Hamilton depression rating scale (HDRS) and the Hamilton anxiety rating scale (HAMA) were used to measure the degree of depression and anxiety, respectively. For this study, no physical symptoms from HAMD (pure HAMD) and HAMA (pure HAMA) were considered to calculate only the scores for pure depression and anxiety. Using HAMD, we created two subdomains: physiosomatic HAMD, which included somatic anxiety, gastrointestinal (GIS) anxiety, genitourinary anxiety, and hypochondriasis; and pure depression HAMD: depressed mood, guilt feelings, suicidal thoughts, and loss of interest. The HAMA was also divided into two subdomains: physiosomatic HAMA—somatic sensory, cardiovascular, genitourinary, and autonomic symptoms, as well as GIS; pure anxiety HAMA—anxious mood, tension, fears, anxiety, and anxious behavior during the interview [39]. Additionally, a pure FibroFatigue (FF) score was calculated by adding the following items to the FF scale: muscular pain, muscle tension, fatigue, autonomous symptoms, GIS, headache, and flu-like malaise [39,40,41,42]. qSUM was also evaluated, representing the total score from the questionnaires. Scores on the HAMA were interpreted using established severity thresholds: 0–7 indicated no or minimal anxiety, 8–14 for mild anxiety, 15–23 for moderate anxiety, and ≥24 indicated severe anxiety [43]. For the 17-piece HAMD, we applied commonly used cut-off values defining depressive symptoms’ severity: 0–7 no depression (remission), 8–16 for mild depression, 17–23 for moderate depression, and ≥24 indicating severe depression, as reported in large clinical validation studies [44,45]. These cut-offs were used for a descriptive characterization of symptom severity. Concerning the FF questionnaire, as no universally established clinical cut-off values exist, scores were analyzed as continuous variables. Descriptive severity bands reported in previous works (mild ≈ 0–20, moderate ≈ 21–40, severe > 40) were used only for contextual interpretation [40,46].

### 2.5. Sample Collection and Processing

Specimens used in this study were whole peripheral blood (6 mL) and serum (5 mL). Blood samples were collected into sodium heparinised collection tubes between 9 a.m. and 12 a.m. and processed within 12 h of collection.

### 2.6. Cytokine Quantification

For the study of cytokine profiles, the Cytometric Bead Array Human Th1/Th2/Th17 Cytokine Kit (BD™, San Jose, CA, USA, #560484) was used (Table S1), which allows the simultaneous measurement of single serum samples of Interleukin-2 (IL-2), Interleukin-4 (IL-4), Interleukin-6 (IL-6), Interleukin-10 (IL-10), Tumor Necrosis Factor (TNF), Interferon-gamma (IFN-gamma), and Interleukin-17A (IL-17A). The acquisition of the samples was performed on FACS Canto II (BD™, San Jose, CA, USA), using the FACS Diva software v.9.0. Results were analyzed using the FCAP Array™ v3.0 software (Softflow, Inc., Pécs, 91/B Pellérdi út, Hungary). All values below the OOR were calculated as LOD/2.

To summarize multiple related immune parameters into a single metric, we calculated a composite z-score. Each variable was standardized by subtracting the sample mean and dividing by the standard deviation. Variables were reversed as necessary so that higher values consistently indicated higher immune activation. The standardized scores were then averaged across variables to create a single composite score for each participant [47,48]. The composite score was calculated using formulas presented in Table 1.

### 2.7. Multicolor Phenotyping Analysis of Surface Markers

The lymphocytes (CD45^+^), T cells (CD3^+^), helper T cells (CD4^+^), cytotoxic T cells (CD8^+^), B-cells (CD19^+^) and NK cells (CD16 ^+^ CD56^+^) were determined using BD Multitest 6-Color TBNK with BD Trucount Tubes (BD Bioscience, San Jose, CA, USA, cat.# 337166), according to the manufacturer’s instructions. For each test, 50 µL of whole blood was used. Stained samples were acquired immediately on a FACS Lyric (BD Biosciences) using FACS Suite Clinical software (BD Biosciences, San Jose, CA, USA).

Gating strategy: Lymphocytes were defined on a CD45 vs. SSC plot. The proportions of basic lymphocyte subsets (T, B, NK) were determined after plotting CD3 vs. CD19, CD3 vs. CD56 + CD16, CD3 vs. CD4 and CD3 vs. CD8 expression, respectively.

### 2.8. Statistical Analysis

GraphPad Prism software ver. 8.0. was used for statistical data processing. Normality of data distribution was assessed using the Shapiro–Wilk test. For non-normally distributed data, results are presented as median with Q1–Q3 ranges. Analyses included the Kruskal–Wallis test for age, Chi^2^ test for sex distribution and Spearman’s correlation. To account for differences in age between groups, we performed rank-transformed ANCOVA: outcome values were rank-transformed and entered into a general linear model with group as the main factor and age as the covariate. For multiple comparisons, we applied the False Discovery Rate (FDR) correction using the Benjamini–Hochberg algorithm. FDR correction was performed separately for each family of related tests (e.g., all cytokines measured in sera). Post hoc power analyses were performed and indicated that all parameters had sufficient statistical power (≥0.8) to detect the observed differences. These analyses support the study being adequately powered for the detected effects, although subtle differences between ME/CFS and LC groups cannot be completely ruled out. Adjusted *p*-values below 0.05 were considered statistically significant.

## 3. Results

Distribution in terms of sex between the three groups was comparable and is presented in Table 2. Age distribution in CFS and LC groups was comparable. The healthy subjects’ mean age was statistically lower than for the two other groups, and this has been acknowledged as a limitation.

### 3.1. Flow Cytometry Results

**Figure 1:**
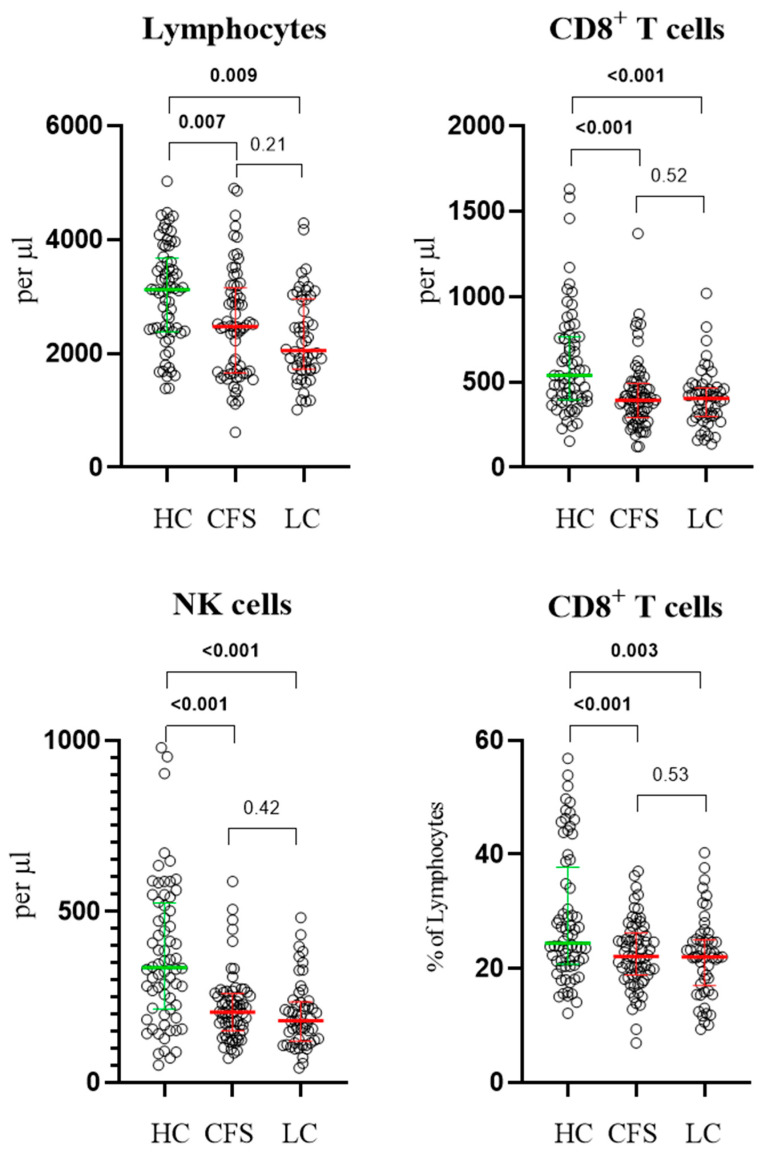
Results from age-adjusted ANCOVA test, showing distribution of lymphocytes, NK and CD8^+^ T cells in the three studied groups as individual points (circles). Exact *p* values are provided. Results are presented as median values and interquartile ranges (coloured lines).

In terms of lymphocytes and lymphocyte subsets, both CFS and LC patients showed significantly lower values compared to HC. In the CFS group, total lymphocyte count (2.472 × 10^9^/L, 1.662–3.155), CD8^+^ T cells (0.394 × 10^9^/L, 0.291–0.492), and NK cells (0.205 × 10^9^/L, 0.151–0.259) were reduced (Figure 1, Table 2). Similarly, in the LC group, lower mean values were observed for total lymphocyte count (2.051 × 10^9^/L, 1.725–2.961), CD8^+^ T cells (0.404 × 10^9^/L, 0.298–0.466), and NK cells (0.180 × 10^9^/L, 0.121–0.235) (Figure 1, Table 3). No significant differences were detected between the CFS and LC groups for these biomarkers.

### 3.2. Cytokine Levels

**Figure 2:**
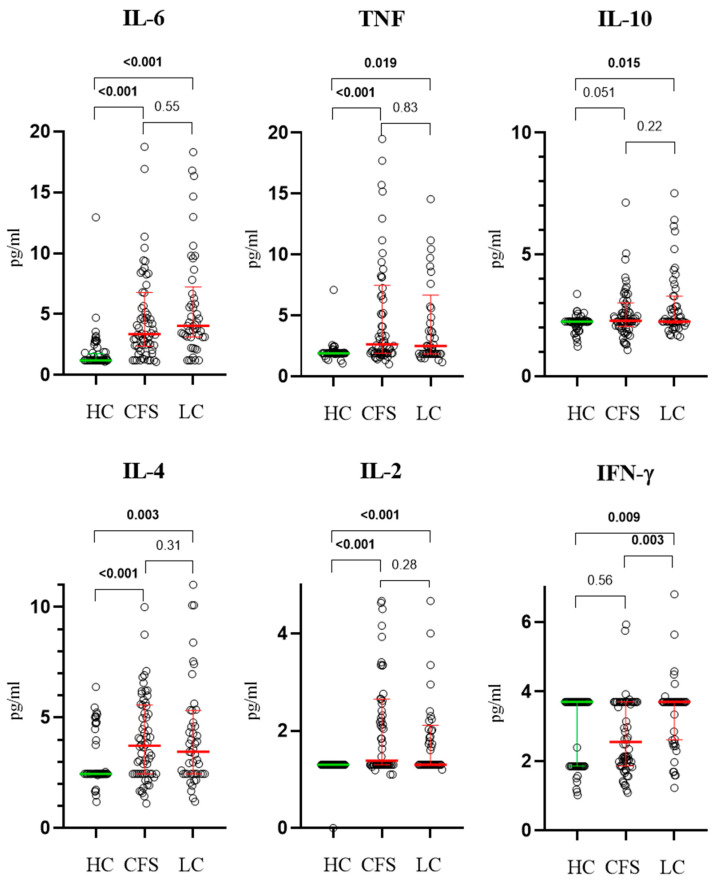
Results from age-adjusted ANCOVA test, showing serum levels of IFN-γ, TNF, IL-4, IL-2, IL-10, and IL-6 in the three studied groups as individual points (circles). Exact *p* values are provided where statistical significance was established. Results are presented as median values and interquartile ranges (coloured lines).

In the CFS group, serum concentrations of TNF (2.64 pg/mL, 1.9–7.47), IL-10 (2.29 pg/mL, 2.05–3.01), IL-6 (3.35 pg/mL, 2.36–6.77), IL-4 (3.72 pg/mL, 2.45–5.57), and IL-2 (1.38 pg/mL, 1.30–2.65) were significantly elevated compared to HC (Figure 2), except for IL-17A (13.75 pg/mL, 10.19–36.51), which did not show any statistically significant difference between groups, and IFN-γ (2.55 pg/mL, 1.85–3.7), which was significantly lower compared to LC. LC patients demonstrated a similar trend, with all cytokines elevated significantly compared to HC. Again, no significant differences were found between CFS and LC patients.

### 3.3. Z-Scores

**Figure 3:**
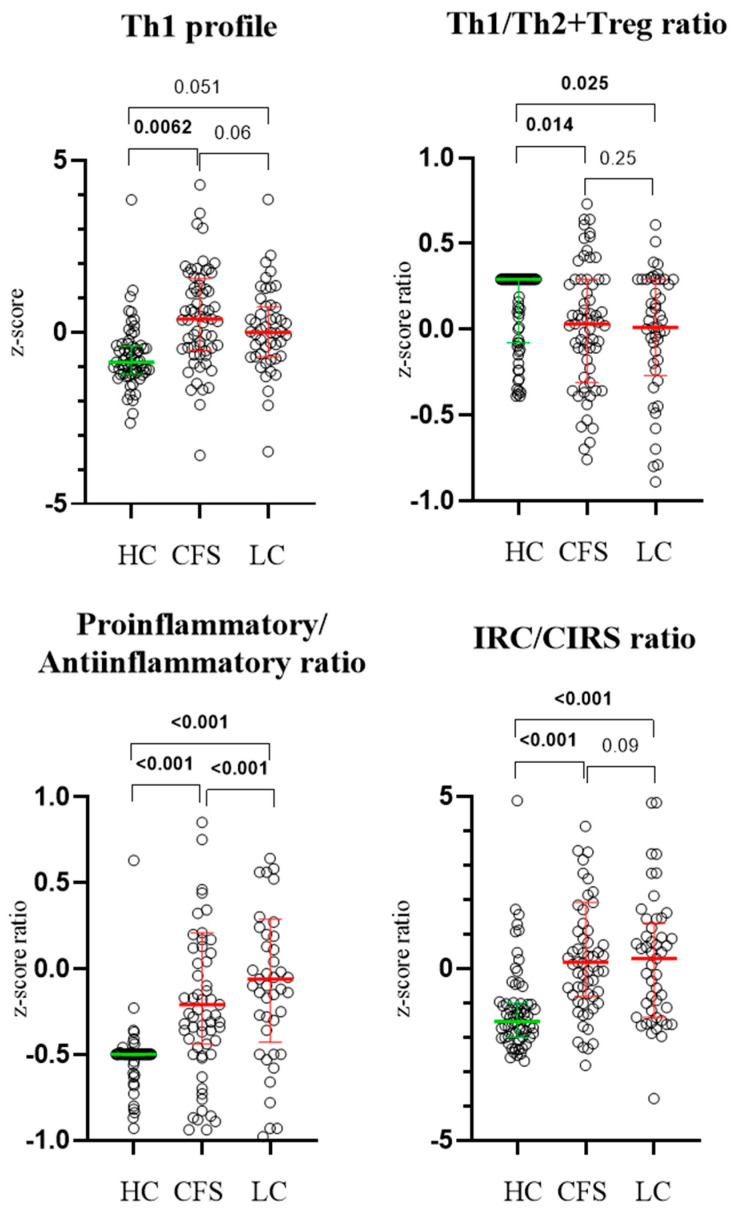
Results from age-adjusted ANCOVA test, showing z-score ratios of Th1 ctokine profile, Th1/Th2 + Treg ratio, proinflamatory/antiinflammatory profile, and IRC/CIRS ratio in the three studied groups as individual points (circles). Exact *p* values are provided where statistical significance was established. Results are presented as median values and interquartile ranges (coloured lines).

Analysis revealed that the Th1/Th2 + Treg z-score ratio was significantly higher in LC patients compared to HC (Figure 3). CFS patients also displayed elevated Th1/Th2 and Th1/Th2 + Treg ratios. No significant z-score differences were found between CFS and LC groups. Increased Th1 profile, Th1/Th2 ratio, and Th1/Th2 + Treg ratio were also observed, based on both serum cytokine concentrations and mean fluorescence intensity (MFI) from flow cytometry analysis (Table 4).

### 3.4. Rating Scales

Anxiety, depressive, and fatigue-related symptoms were assessed using the HAM-A, the 17-item HAMD, and the FibroFatigue scale. Median HAM-A scores were 6 (95% CI: 4.22–8.67) in HC, 10 (9.76–12.31) in ME/CFS, and 11 (10.56–13.18) in LC participants; the commonly used clinical cut-off for mild anxiety is 8. ANCOVA, adjusted for age, indicated a significant group effect (Table 5).

Median HAMD scores were 7.5 (6.04–11.74) in HC, 14 (12.21–15.49) in ME/CFS, and 13 (11.51–15.41) in LC; the standard cut-off for mild depression used was 8. The adjusted ANCOVA test showed a significant difference among groups (*p* = 0.037).

FF scores indicated more pronounced fatigue and fibromyalgia-like symptoms in patient groups, with medians of 8 (6.23–13.77) in HC, 22 (20.74–24.68) in ME/CFS, and 26 (24.43–29.40) in LC; although no universally validated cut-off exists, a previously suggested threshold for severe symptom burden is 21. The main differences occurred between patient groups (CFS and LC) compared to HC. No statistically significant differences were detected between CFS and LC groups.

### 3.5. Correlation Analysis

In the CFS group, positive correlations were observed between CD8 numbers and IL-6, IL-10 and IL-4, IL-10 and IL-6, IL-6 and TNF, and IL-2 and IFN-γ serum concentrations (Table 6). Negative correlations included total lymphocyte count with IFN-γ, CD19 numbers with Th1 MFI, and CD19 numbers with Th1/Th2 + Treg MFI. Furthermore, lymphocyte subsets negatively correlated with FF scores and partially with HAMA/HAMD scores (Table 7).

In the LC group, positive correlations were found between CD56 numbers and IL-2, IL-10 and HAMD (Table 8 and Table 9), IL-6 and HAMD, total lymphocyte count and HAMA, total lymphocyte count and HAMD, CD19 numbers and HAMA, IL-6 and IL-10, IL-4 and IL-10, and IL-6 and IL-4. Negative correlations were detected between CD8 numbers and IL-6, and IL-2 with HAMA scores (Table 8).

## 4. Discussion

This study shows that ME/CFS and Long COVID exhibit remarkably similar immune alterations. Using ANCOVA to adjust for age differences, we found no significant distinctions between the two syndromes in almost any of the examined lymphocyte subsets or cytokine biomarkers. These results suggest overlapping immunological chnages and support the possibility of common underlying mechanisms in both conditions. The prominent low lymphocyte numbers documented in this study might be a result of chronic immune activation caused by viral infections, leading to immune exhaustion, a phenomenon depicted before [49,50,51,52,53]. On the other hand, the presence of an underlying infection, combined with low NK and CD8^+^ T cells with possible impaired function in viral defense, may be one of the reasons why patients report frequent common viral infections, causing the flu-like, GI symptoms typical for ME/CFS [54]. Research conducted in the 1990s found that there was an increase in CD8^+^ T cells [55,56], but more recent research shows that there was no discernible change in the total number of cytotoxic T cells [18,19,50]. On the other hand, dysfunctional or, more accurately, clonally exhausted CD8 T cells have been reported. They are characterized by diminished proliferation, aberrant metabolic processes, persistently high levels of inhibitory receptor expression (PD-1, CTLA-4, Lag-3, TIGIT, etc.), gradual loss of effector functions (IL-2, IFNγ, TNF), etc. [57,58]. This kind of T cell dysfunction, which is brought on by ongoing antigen stimulation, is frequently observed in cases of chronic viral infections.

Beyond function, the number of NK cells can also indicate underlying immune dysregulation. Several studies have shown increased numbers of NK cells in ME/CFS patients, albeit with compromised functionality [55,59,60]. This paradoxical increase suggests a compensatory response to chronic immune activation or infection. Similar findings have been observed in Long COVID patients, where an increase in NK cell numbers has been documented [20]. This similarity further underscores the overlapping immune features between the two conditions. However, there are studies where no differences in terms of base lymphocyte subsets were observed when comparing COVID-19 survivors with and without Long COVID symptoms. This observation may reflect that Long COVID may be driven more by post-infectious immune dysregulation, metabolic or autonomic disturbances, or persistent inflammatory processes, rather than by initial quantitative immune suppression [61]. More in-depth immune cell phenotyping is needed to point toward potential more unambiguous conclusions.

The increased CD4^+^/CD8^+^ ratio observed in both groups, compared to HC, could potentially serve as a marker for disease severity and could be used for monitoring therapy effectiveness. This statement can be supported by the observed negative correlation between CD4/CD8 ratio and the qSUM z-score. An early meta-analysis shows a link between depression, commonly observed in CFS/ME patients [62,63,64,65], and an increased CD4^+^/CD8^+^ ratio [66].

Abnormal cytokine profiles have been widely reported, although findings vary. Some studies link these cytokine levels to symptom severity, particularly during early ME/CFS stages [67]. Several researcher groups have documented that proinflammatory cytokines are elevated in both ME/CFS patients [68,69,70,71] and Long COVID patients [72,73,74]. Similar findings (similar for both groups) have also been reported in terms of increased numbers of activated CD8^+^ cytotoxic cells [20,56,60,73,75,76,77].

The presence of chronic low-grade inflammation may be a potential cause of the symptoms characteristic of ME/CFS, including musculoskeletal pain and persistent fatigue [78]. The presence of low-grade inflammation in ME/CFS is determined by the predominance of the Th1 cytokine profile, which was also seen in the patients in our study and has been documented by other researchers [79]. Other studies report a dysregulated Th response, consisting of both pro- and anti-inflammatory cytokines [26,80,81,82]. Furthermore, earlier work led to the hypothesis that the cytokine profile in ME/CFS patients might correlate to the disease stage—during the acute phase, a Th1 response is predominant, while Th2 response is seen in the remission stage [83]. The Th1 skewed immune response, involving IRS in the ME/CFS and LC patients in our study, might be a result of a more acute stage of the syndrome. Conducting longitudinal studies where disease stages are correlated with cytokine production is important to prove this theory. The observed altered Th1/Th2 + Treg ratio suggests not only an inflammatory Th1 response, but also a possible dysfunction of Tregs, which are essential for maintaining immune tolerance and preventing autoimmunity [84].

The pathogenesis of depression in numerous psychiatric disorders is characterized by chronic inflammation, which is associated with elevated levels of proinflammatory cytokines (such as TNF and IL-6). These cytokines can contribute to the activation of the hypothalamic–pituitary–adrenal (HPA) axis, leading to the release of corticotropin-releasing hormone (CRH) and adrenocorticotropic hormone (ACTH). The activation of the HPA axis can result in increased cortisol production, which may reduce serotonin and catecholamine levels, thereby contributing to the clinical presentation of depression [85]. It is also important to note the correlation between IL-6 and the development of depression in other physical diseases, including breast cancer, ovarian cancer, metastatic cancer, colorectal cancer, cardiovascular disease and rheumatological diseases [86]. Moreover, IL-6 has been demonstrated to reduce the expression of endothelial tight junction proteins, by increasing the permeability of the blood–brain barrier. This allows immune cells to infiltrate the central nervous system, leading to the generation of neuroinflammation [87].

Although there is past research documenting the relationship between cytokines and major depressive disorder and anxiety, this interplay has not been established well enough in the context of CFS/ME and LC [68,88,89,90,91].

In accordance with the data about the pathogenesis of depression discussed above, our study demonstrated positive correlations between IL-6, IL-10 and psychological grading scales (HAMA, HAMD), suggesting that these cytokines may reflect neuropsychological symptoms such as anxiety and depression in LC patients [92,93]. The negative correlation between CD8 T cells and IL-6, and IL-2 with HAMA score, suggests that higher levels of CD8 T cell and lower IL-6 levels may be protective against severe neuropsychological symptoms. These findings may point to potential therapeutic targets, such as IL-6 inhibitors or therapies aimed at boosting CD8 T cell function, to alleviate neuropsychological symptoms in LC patients. The negative correlation between lymphocyte counts and IFN-γ, together with the qSUM score (a potential measure of symptom severity), suggests that higher symptom severity may be associated with lower total lymphocyte levels, reflecting broader immune dysregulation.

A significant body of research exists on the pathogenesis and management of ME/CFS. If Long COVID is shown to be a similar chronic illness with overlapping clinical features and symptoms, it is possible that the existing knowledge on ME/CFS could be beneficial for treating Long COVID patients. Given the prevalence rates reported in early studies on Long COVID, millions of individuals could potentially benefit from these insights into treatment and management. Conversely, the growing public interest in Long COVID and the surge in research efforts might uncover new findings that could also aid ME/CFS patients.

The lack of significant differences in tested biomarkers between ME/CFS and LC raises the question of whether there is a possibility for these conditions to share a common immunopathological basis. In addition, there are similarities in clinical presentation in terms of persistent flu-like symptoms, myalgia, post-exertional fatigue and cognitive dysfunction. Therefore, SARS-CoV-2 could be considered a potential etiological agent associated with ME/CFS, among other associated viruses [8,9,94].

## 5. Conclusions

This study underscores the significant immune dysregulation present in both ME/CFS and Long COVID, with similar reductions in lymphocyte counts and CD8^+^ T cell numbers and elevations in proinflammatory cytokines (IFNγ, TNF, IL-6) correlating with mental health status. The immune similarities between the two conditions may suggest shared points in immune pathways and maybe a common pathophysiology, potentially linking SARS-CoV-2 infection to the onset of ME/CFS-like symptoms. Further research is needed to elucidate the full extent of these immune alterations and their implications for treatment. Therapeutic strategies aimed at restoring immune balance, such as enhancing Treg function or targeting cytokine profiles, may offer relief for both patient groups.

This study has several limitations. First, the control group was younger than the patient groups, which may have influenced immune parameters despite statistical adjustments for age. Second, the cross-sectional design precludes any conclusions about causality between immune alterations and disease status. Third, although most comparisons were adequately powered, the sample sizes for some subgroup analyses, such as ME/CFS versus LC, were relatively small, and subtle differences may have been missed due to limited statistical power (risk of Type II error). We also acknowledge that laboratory assay variability may have influenced some biomarker measurements, despite standardized procedures. Future studies using harmonized protocols across multiple centers are needed to validate and extend these findings. These limitations should be considered when interpreting the results, and future studies with larger, age-matched cohorts and longitudinal designs are warranted.

## Figures and Tables

**Table 1:** Cytokine profiles and composite z-score formulas used for their calculations.

Cytokine Profile	Formula
Th1 profile	zIL-2 + zIFN-γ
Th1/Th2 ratio	z(zIFN-γ + zIL-2) − zIL-4
Th1/Treg ratio	z(zIFN-γ + zIL-2) − zIL-10
Th1/Th2 + Treg ratio	z(zIFN-γ + zIL-2) − z(zIL-4 + zIL-10)
Th17 profile	zIL-6 + zIL-17A
Proinflammatory cytokine profile	zIL-2 + zIL-6 + zIFN-γ + zTNF
Anti-inflammatory cytokine profile	zIL-4 + zIL-10
Proinflammatory/Anti-inflammatory cytokine ratio	z(zIL-2 + zIL-6 + zIFN-γ + zTNF) − z(zIL-4 + zIL-10)
Inflammatory Response System (IRS)	zIL-6 + zTNF-α + zIL-2 + zIFN-γ + zIL-17A
Compensatory Inflammatory Response System (CIRS)	zIL-4 + zIL-10
All IRS/all CIRS ratio	z(zIL-2 + zIL-6 + zTNF + zIFN-γ + zIL-17A) − z(zIL-4 + zIL-10)

**Table 2:** Descriptive statistics of participants, categorized as CFS patients, LC and HC, containing information about age and sex distribution within the groups.

Column1	HC	CFS	LC	*p* Value
**N**	70	65	54	
Male, N(%)	27 (39%)	20 (31%)	11 (20%)	0.093
Female, N(%)	43 (61%)	45 (69%)	43 (80%)	0.093
Age, N ± SD	31.60 ± 9.6	45.19 ± 13.4	51.2 ± 10.6	<0.001

**Table 3:** Descriptive statistics of flow cytometry data from CFS, LC and HC groups, containing median values, 95% CI, and ANCOVA *p*-values, adjusted for age.

Biomarkers	Group	N	Median	Actual Confidence Level	Lower CI Limit	Upper CI Limit	ANCOVA *p*-Value (Adjusted for Age)
Lymphocytes (×10^9^/L)	HC	70	3.123	96.15%	2.671	3.342	0.002
	CFS	65	2.472	96.15%	2.345	2.851	
	LC	54	2.051	95.98%	1.910	2.457	
CD8^+^ cells (×10^9^/L)	HC	70	0.538	96.15%	0.464	0.641	<0.001
	CFS	65	0.394	96.15%	0.357	0.429	
	LC	54	0.404	95.98%	0.339	0.443	
NK cells (×10^9^/L)	HC	70	0.336	96.15%	0.288	0.408	<0.001
	CFS	65	0.205	96.15%	0.178	0.229	
	LC	54	0.180	95.98%	0.157	0.213	
CD8^+^ cells (% from lymphocytes)	HC	70	24.44	96.15%	23.41	27.96	<0.001
	CFS	65	22.13	96.15%	20.22	24.6	
	LC	54	22	95.98%	21.1	23.3	

**Table 4:** Descriptive statistics of cytokine profile data from CFS, LC and HC groups, containing median values, 95% CI, and ANCOVA *p*-values, adjusted for age.

Biomarkers	Group	N	Median	Actual Confidence Level	Lower CI Limit	Upper CI Limit	ANCOVA *p*-Value (Adjusted for Age)
Th1 profile (MFI)	HC	70	−0.88	96.44%	−1.08	−0.6	0.008
	CFS	65	0.38	95.02%	−0.11	0.74	
	LC	54	−0.01	95.56%	−0.36	0.35	
Th1/Th2 ratio (MFI)	HC	70	−0.96	96.44%	−1.13	−0.61	<0.001
	CFS	65	0.5	95.02%	0.09	0.67	
	LC	54	0.06	95.56%	−0.44	0.36	
Th1/Th2 + Treg ratio (MFI)	HC	70	−0.87	96.44%	−1.13	−0.48	0.011
	CFS	65	0.29	95.02%	0.03	0.59	
	LC	54	0.03	95.56%	−0.57	0.43	
Th17 profile (MFI)	HC	70	−0.79	96.44%	−0.92	−0.6	0.014
	CFS	65	−0.22	95.02%	−0.52	0.03	
	LC	54	−0.24	95.56%	−0.45	0.18	

**Table 5:** Descriptive statistics of clinical rating scale data from CFS, LC and HC groups, containing median values, 95% CI, and ANCOVA *p*-values, adjusted for age.

Scale	Group	N	Median	Actual Confidence Level	Lower CI Limit	Upper CI Limit	Cut-Off Value	ANCOVA *p*-Value (Adjusted for Age)
HAMA	HC	70	6	96.91%	4.219	8.670	8	<0.001
	CFS	65	10	96.86%	9.755	12.31		
	LC	54	11	96.04%	10.56	13.18		
HAMD	HC	70	7.5	96.91%	6.040	11.74	8	0.038
	CFS	65	14	96.86%	12.21	15.49		
	LC	54	13	96.04%	11.51	15.41		
FF	HC	70	8	96.91%	6.232	13.77	21	<0.001
	CFS	65	22	96.86%	20.74	24.68		
	LC	54	26	96.04%	24.43	29.40		

**Table 6:** Correlation matrix containing data from CFS patients. *—Strong positive correlation; **—moderate positive correlation; ***—moderate negative correlation; NS — not significant.

	IL-10 (pg/mL)	INF-Y (pg/mL)	TNF (pg/mL)	IL-4 (pg/mL)	CD8^+^ Cells (×10^9^/L)	B Cells (×10^9^/L)
IL-10 (pg/mL)	1	NS	NS	0.79 *	NS	NS
IL-6 (pg/mL)	0.64 **	NS	0.73 **	NS	0.28 **	NS
IL-2 (pg/mL)	NS	0.49 **	NS	NS	NS	NS
Lymphocytes (×10^9^/L)	NS	−0.28 ***	NS	NS	NS	NS
Th1 profile (MFI)	NS	NS	NS	NS	NS	−0.28 ***
Th1/Treg ratio (MFI)	NS	NS	NS	NS	NS	−0.28 ***

**Table 7:** Correlation matrix containing data from flow cytometry analysis and rating scales scores for CFS patients; ***—moderate negative correlation.

	FF	HAMA	HAMD
Lymphocytes (×10^9^/L)	−0.31 ***	0.04	0.10
T cells (×10^9^/L)	−0.29 ***	−0.19	−0.25 ***
CD4^+^ cells (×10^9^/L)	−0.28 ***	−0.16	−0.18
CD8^+^ cells (×10^9^/L)	−0.21	−0.19	−0.27 ***
B cells (×10^9^/L)	−0.27 ***	−0.17	−0.13
NK cells (×10^9^/L)	−0.33 ***	−0.29 ***	−0.23

**Table 8:** Correlation matrix, containing cytokine and scales data from LC patients. **—Moderate positive correlation; ***—moderate negative correlation.

	HAMA	HAMD
**IL-10 (pg/mL)**	NS	0.34 **
**IL-2 (pg/mL)**	−0.27 ***	NS
**IL-6 (pg/mL)**	NS	0.37 **
**B cells (×10^9^/L)**	0.28 **	0.32 **
**Lymphocytes (×10^9^/L)**	0.35 **	0.30 **

**Table 9:** Correlation matrix, containing cytokine and flow cytometry data from LC patients. **—Moderate positive correlation; ***—moderate negative correlation.

	IL-4 (pg/mL)	IL-10 (pg/mL)	CD8^+^ (×10^9^/L)	NK Cells (×10^9^/L)
**IL-4 (pg/mL)**	1	0.50 **	NS	NS
**IL-6 (pg/mL)**	0.38 **	0.57 **	−0.31 ***	NS
**IL-2 (pg/mL)**	NS	NS	NS	0.44 **

## Data Availability

Data are contained within the article.

## Supplementary Materials

The following supporting information can be downloaded at: https://www.mdpi.com/article/10.3390/biomedicines13123001/s1, Table S1: Sensitivity and intra-/inter-assay variability of the BD Human Th1/Th2/Th17 CBA kit.
