# Supplementary material for: Comparable Immune Alterations and Inflammatory Signatures in ME/CFS and Long COVID
Source: Biomedicines. 2025 Dec 8;13(12):3001. doi: 10.3390/biomedicines13123001 (PMC12730569; doi:10.3390/biomedicines13123001)
Supplement: Supplementary file 1 [file biomedicines-13-03001-s001.zip › biomedicines-3962999-supplementary.pdf]

Table S1: Sensitivity and intra-/inter-assay variability of the BD Human Th1/Th2/Th17 CBA kit.

Cytokine	Sensitivity (pg/mL)	Intra-assay CV (%)	Inter-assay CV (%)
IL-2	2.6	4–5	7–9
IL-4	4.9	2–5	5–11
IL-6	2.4	4–6	7–13
IL-10	4.5	4–5	6–11
TNF	3.8	6–8	8–12
IFN-γ	3.7	3–4	8–11
IL-17A	18.9	3–5	6–12

** Data derived from the manufacturer's technical manual (BD Biosciences, Human Th1/Th2/Th17 CBA Kit, Cat. No. 560484). Sensitivity corresponds to the theoretical limit of detection (two standard deviations above the median fluorescence of 0 pg/mL standard). CV = coefficient of variation.*
